# Organised Colorectal Cancer Screening and Changes in Mortality and Incidence Trends: A Population-Based Study

**DOI:** 10.3390/cancers18081313

**Published:** 2026-04-21

**Authors:** Astrid Díez-Martín, Margarita Castro, Isolina Santiago, Raquel Almazán, Ángel Gómez-Amorín, Cristina Regueiro-Expósito, Pedro Davila-Piñón, Joaquín Cubiella

**Affiliations:** 1 Grupo de Investigación en Oncología Digestiva-Ourense, Fundación Pública Gallega de Investigación Biomédica Galicia Sur, Ourense, Spain; astrid.diez@iisgaliciasur.es (A.D.-M.); cristina.regueiro@iisgaliciasur.es (C.R.-E.); pedro.davila@iisgaliciasur.es (P.D.-P.); 2 Registro Gallego de Tumores, Dirección Xeral de Saúde Pública, Ourense, Spain; margarita.castro.bernardez@sergas.es; 3 Servizo de Información de Saúde, Dirección Xeral de Saúde Pública, Santiago de Compostela, Spain; mariaisolina.santiago.perez@sergas.es; 4 Servizo de Detección Precoz de Enfermidades, Dirección Xeral de Saúde Pública, Santiago de Compostela, Spain; raquel.almazan.ortega@sergas.es (R.A.); ángel.gomez.amorin@sergas.es (Á.G.-A.); 5 Grupo de Investigación en Oncología Digestiva-Ourense, Fundación Pública Gallega de Investigación Biomédica Galicia Sur, Área Sanitaria de Ourense, Verín e O Barco de Valdeorras, Ourense, Spain

**Keywords:** colorectal cancer, population-based screening, faecal immunochemical test, effectiveness, cancer registry

## Abstract

**Background:** Organised colorectal cancer (CRC) screening programmes have been widely implemented across Europe; however, robust population-level evaluations of their real-world effectiveness, particularly for programmes based exclusively on faecal immunochemical testing (FIT), remain limited. The Galician CRC screening programme was progressively implemented between 2013 and 2019. **Methods:** We conducted a population-based ecological time-series study using data from the Galician Tumour Registry (ICD-10 C18–C21) for 2015–2023. Age-standardised mortality (ASMR) and incidence (ASIR) rates were analysed. They were calculated using the direct standardisation method, applying age-specific rates to the 2013 European Standard Population (ESP2013). Structural changes associated with programme implementation were evaluated using interrupted time-series (ITS) models, estimating annual percent change (APC) before and after implementation and the net change in slope (ΔAPC). Absolute and relative changes in ASMR and ASIR were calculated by comparing 2015–2017 and 2019–2023. Analyses were performed for the overall population and for individuals aged 50–69 years. **Results:** Between 2015 and 2023, overall CRC mortality declined significantly (APC −3.00%; 95% CI −3.37 to −2.63). ITS analysis demonstrated a marked modification of mortality trajectories following programme implementation. Mortality shifted from an increasing pre-implementation slope (APC +13.70%; 95% CI 10.12, 17.39) to a significant annual decline post-implementation (APC −3.62%; 95% CI −4.47, −2.76), yielding a ΔAPC of −17.32. In individuals aged 50–69 years, the structural change was more pronounced (ΔAPC −19.88), with post-implementation mortality decreasing by −8.08% annually (95% CI −10.43, −5.66). Incidence showed a comparable structural modification. Overall APC changed from +15.26% (95% CI 5.48, 25.95) before implementation to −2.48% (95% CI −5.29, 0.41) afterwards (ΔAPC −17.74). In the screening-eligible population, APC shifted from +21.32% (95% CI 4.60, 40.71) to −3.74% (95% CI −7.62, 0.30), corresponding to a ΔAPC of −25.06. Descriptively, ASMR declined from 41.92 to 35.91 per 100,000 (−14.33%), and ASIR from 98.37 to 85.16 per 100,000 (−13.42%) between 2015 and 2017 and between 2019 and 2023. Relative reductions were larger in individuals aged 50–69 years and were more pronounced for colon cancer than for rectal cancer. **Conclusions:** Implementation of an organised FIT-based screening programme was associated with a structural change in CRC mortality and incidence trends, particularly among individuals aged 50–69 years.

## Simple Summary

Colorectal cancer is one of the leading causes of cancer-related death, but it can be prevented or detected early through population screening programmes. In recent years, many regions have introduced organised screening using a stool-based test, yet evidence on its real-world impact at the population level remains limited. In this study, we evaluated the introduction of a regional screening programme in Galicia (Spain) using data from a population-based cancer registry. We analysed trends in cancer incidence and mortality before and after the programme was implemented. We observed a clear change in trends, with reductions in both incidence and mortality, particularly among individuals invited to screening. These findings suggest that organised screening programmes can be associated with measurable changes at the population level and support their continued implementation and optimisation in public health systems.

## 1. Introduction

Colorectal cancer (CRC) represents a major public health challenge in the Western world, and particularly in Spain, where it is the most frequently diagnosed malignancy and the second leading cause of cancer-related death. However, its natural history—characterised by slow progression from precursor lesions and a prolonged asymptomatic phase—provides a critical window of opportunity for early detection and for interrupting the adenoma–carcinoma sequence before the disease progresses to advanced, life-threatening stages [1].

The implementation of population-based CRC screening programmes has been underpinned by robust evidence derived from randomised controlled trials conducted in Western populations. Early landmark studies based on guaiac faecal occult blood testing (gFOBT) demonstrated that screening significantly reduces CRC-specific mortality, with relative reductions ranging from approximately 16% in intention-to-treat analyses to up to 33% among adherent participants. However, these trials did not demonstrate a short-term reduction in CRC incidence [2,3,4].

The introduction of the faecal immunochemical test (FIT) substantially improved diagnostic performance, offering higher sensitivity for advanced neoplasia and enabling quantitative threshold adjustment. Observational population-based studies have reported substantial reductions in CRC mortality and, in some settings, incidence following organised FIT-based screening [5,6]. Large longitudinal population studies in Italy and the United States have reported mortality reductions exceeding 50% among highly adherent participants [6,7,8]. Similarly, national experience in Taiwan has confirmed these benefits in Asian populations, showing a 35% reduction in CRC mortality [9]. These analyses have also documented reductions in CRC incidence, ranging from 10% to 25%, likely reflecting the detection and removal of precursor lesions [10].

A recent global systematic review and meta-analysis of 58 organised CRC screening programmes across 22 countries reported an overall 41.8% reduction in CRC-related mortality associated with organised screening [11]. However, the majority of included programmes were originally based on guaiac faecal occult blood testing (gFOBT) or mixed modalities, and only a minority were implemented as exclusively FIT-based strategies from inception. Moreover, FIT-specific mortality reductions were strongly influenced by programme duration, with limited statistical significance observed in programmes with shorter follow-up. These findings highlight the need for long-term, real-world evaluations of organised FIT-based screening programmes using high-quality population registry data.

To address this evidence gap, we evaluated the population-level impact of the Galician Colorectal Cancer Early Detection Programme (PGDPCC) [12], an organised FIT-based screening programme implemented asynchronously across healthcare areas. This regional implementation reflects the decentralised structure of the Spanish healthcare system, in which population-based screening programmes are organised and delivered by the Autonomous Communities, while the Ministry of Health provides regulatory oversight and ensures coordination and equity at the national level.

Using population-based cancer registry data, we analysed trends in colorectal cancer mortality and incidence and formally assessed structural changes in temporal trajectories following programme implementation.

## 2. Materials and Methods

### 2.1. Study Design and Setting

We conducted a retrospective, population-based observational study to evaluate temporal changes in colorectal cancer (CRC) mortality and incidence following the implementation of the organised population-based screening programme (PGDPCC) in Galicia, Spain. The study period extended from 1 January 2015 to 31 December 2023, enabling assessment of epidemiological trends after the phased roll-out of the programme.

The PGDPCC is an organised screening programme based on biennial faecal immunochemical testing (FIT), using a threshold of 20 μg haemoglobin per gram of faeces, targeting individuals aged 50–69 years. This threshold was uniformly defined at the national level in Spain, in accordance with coordinated public health recommendations, ensuring consistency across regional screening programmes. Implementation occurred asynchronously across the seven healthcare areas between 2013 and 2019.

This study represents an analysis of routinely collected public health data derived from established administrative and cancer registry databases. All data were fully anonymised prior to analysis, and no individual-level identifiers were accessible to the investigators. In accordance with Spanish legislation, including Law 14/2007 of 3 July on Biomedical Research, and European data protection regulations (Regulation (EU) 2016/679, GDPR; Recital 26), studies based on fully anonymised data that cannot be linked to identifiable individuals do not require formal research ethics committee approval or informed consent.

### 2.2. Study Population and Data Sources

The study covered the entire Autonomous Community of Galicia. Population denominators were obtained from the official regional health insurance registry (SIAC). CRC mortality and incidence data were obtained from the Galician Tumour Registry (REGAT) [13], a cancer population-based registry. All malignant neoplasms coded as ICD-10 C18–C21 diagnosed or recorded between 2015 and 2023 were included. REGAT provided annual age-standardised mortality rates (ASMR) and age-standardised incidence rates (ASIR), expressed per 100,000 inhabitants. Rates were available stratified by age group (<50, 50–69, ≥70 years), sex, tumour location (colon and rectum), and healthcare area.

### 2.3. Outcomes

The primary outcomes were ASMR and ASIR for CRC, expressed per 100,000 inhabitants.

### 2.4. Statistical Analysis

#### Interrupted Time-Series Analysis

The primary analytical approach was interrupted time-series (ITS) analysis using segmented log-linear regression. This quasi-experimental method allows estimation of changes in outcome trajectories following the introduction of a population-level intervention while accounting for underlying secular trends.

Given the asynchronous roll-out of the PGDPCC, pre- and post-implementation periods were defined separately for each healthcare area. To reflect the expected delay between screening exposure and measurable population-level impact, a two-year latency period following programme initiation was incorporated into the definition of the post-implementation phase (Supplementary Table S1). This lag was defined a priori as an operational assumption to account for the time required for invitation, test completion, diagnostic colonoscopy after a positive FIT, and subsequent clinical management before changes in annual incidence and mortality rates become observable.

For each area and cohort, annual age-standardised rates were modelled as a function of time, with parameters representing the baseline pre-implementation trend and the change in slope after implementation. From these segmented models, the annual percent change (APC) was estimated for both pre- and post-implementation periods, calculated as:

APC = (e^β^ − 1) × 100.

The net change in slope (ΔAPC) was defined as the difference between post- and pre-implementation APC estimates and interpreted as a measure of structural change in trend.

Area-specific APC estimates were aggregated at regional level using population-weighted averages. Statistical inference was restricted to periods with at least three annual observations to ensure stability of slope estimation. Because ΔAPC represents the difference between correlated slope estimates derived from the same segmented model, formal confidence intervals were not calculated for this metric; it was interpreted descriptively alongside the statistical significance of the pre- and post-implementation slopes.

Model assumptions were evaluated by inspection of residual plots and assessment of first-order autocorrelation. Given the limited number of annual observations within several pre- and post-implementation segments, formal autoregressive correction was not undertaken, as it would have resulted in unstable and potentially overparameterized models. Consequently, results were interpreted conservatively.

### 2.5. Overall Secular Trend Analysis

In addition to the ITS approach, we summarised overall secular trends across 2015–2023 using log-linear models to provide a global APC estimate independent of programme timing, as a descriptive complement to the segmented ITS results. These models characterised the general temporal evolution of CRC mortality and incidence and were interpreted in conjunction with the ITS results.

### 2.6. Absolute and Relative Changes in Age-Standardised Rates

To contextualise the magnitude of changes at the population level, complementary descriptive analyses compared two aggregated periods: a pre-screening reference period (2015–2017) and a post-implementation period (2019–2023). The year 2018 was considered transitional and excluded from these comparisons to minimise contamination by partial implementation effects.

For each stratum, we computed the mean annual ASMR/ASIR in 2015–2017 and 2019–2023. Confidence intervals of 95% (95% CI) for period means and for absolute differences were derived using t-distribution methods based on the annual observations within each period; relative changes were expressed as percentage variation relative to the pre-period mean.

### 2.7. Stratified and Exploratory Analyses

All analyses were conducted for the overall population and separately for individuals aged 50–69 years. Stratified analyses by sex, age group, and tumour location were performed to assess consistency and specificity of observed effects. Given the small number of healthcare areas, the participation–impact association was assessed using Spearman’s rank correlation (two-sided α = 0.05).

### 2.8. Statistical Software

All statistical analyses were performed using IBM SPSS Statistics version 25.0 (IBM Corp., Armonk, NY, USA). Two-sided *p*-values < 0.05 were considered statistically significant.

## 3. Results

### 3.1. Area-Level Analysis

Segmented regression models incorporating area-specific implementation dates revealed heterogeneity in post-implementation trends across healthcare areas (Supplementary Table S2). In the post-implementation period, significant declines in CRC mortality were observed in A Coruña, Santiago, Ourense, Pontevedra, and Vigo. In Ferrol and Lugo, mortality slopes were negative but did not reach statistical significance. In the screening-eligible population (50–69 years), post-implementation mortality reductions were more pronounced, particularly in A Coruña (APC −14.27%; 95% CI −17.57 to −10.85), Lugo (−10.68%; 95% CI −19.65 to −1.10), Vigo (−7.96%; 95% CI −14.90 to −0.46), and Santiago (−4.78%; 95% CI −8.80 to −0.59). Post-implementation incidence slopes were more variable and did not consistently achieve statistical significance across areas.

### 3.2. Aggregated Regional ITS Analysis

Before programme implementation, overall CRC mortality exhibited a statistically significant increasing trend (APC +13.70%; 95% CI 10.12 to 17.39). Given the limited number of annual observations in some pre- and post-implementation segments, particularly in healthcare areas with later programme rollout, slope estimates should be interpreted cautiously and are presented as descriptive indicators of temporal trends. Following implementation, this trend reversed to a statistically significant decline (APC −3.62%; 95% CI −4.47 to −2.76), corresponding to a net change in slope (ΔAPC) of −17.32 percentage points. In the screening-eligible population (50–69 years), the structural change was more pronounced (ΔAPC −19.88), with slopes shifting from +11.80% (95% CI 1.86 to 22.72) before implementation to −8.08% (95% CI −10.43 to −5.66) thereafter (Table 1, Supplementary Figure S1).

Among individuals aged ≥ 70 years, mortality also shifted from a marked pre-implementation increase (APC +20.35%; 95% CI 14.42 to 26.60) to a significant post-implementation decline (−2.22%; 95% CI −3.40 to −1.02), yielding ΔAPC −22.57. In contrast, among individuals aged < 50 years, no consistent reversal of trend was observed; estimates were imprecise and slopes did not demonstrate clear structural modification.

Sex-specific analyses demonstrated a substantial structural change in men (ΔAPC −20.99), with slopes shifting from +17.59% to −3.40%. In women, although the pre-implementation slope was positive but not statistically significant (+7.55%; 95% CI −1.54 to 17.49), the post-implementation decline was significant (−3.41%; 95% CI −5.47 to −1.31), yielding ΔAPC −10.97. By tumour location, both colon and rectal cancers demonstrated reversal of slope following programme implementation. The structural change was slightly greater for colon cancer (ΔAPC −14.94) than for rectal cancer (ΔAPC −15.19), consistent with the expected biological performance of FIT-based screening.

For incidence, the overall APC changed from +15.26% (95% CI 5.48 to 25.95) to −2.48% (95% CI −5.29 to 0.41), with ΔAPC −17.74. Among individuals aged 50–69 years, ΔAPC reached −25.06, reflecting a marked modification in incidence trajectory after programme implementation (Table 1, Supplementary Figure S2).

### 3.3. Overall Secular Trend Analysis (2015–2023)

**Figure 1:**
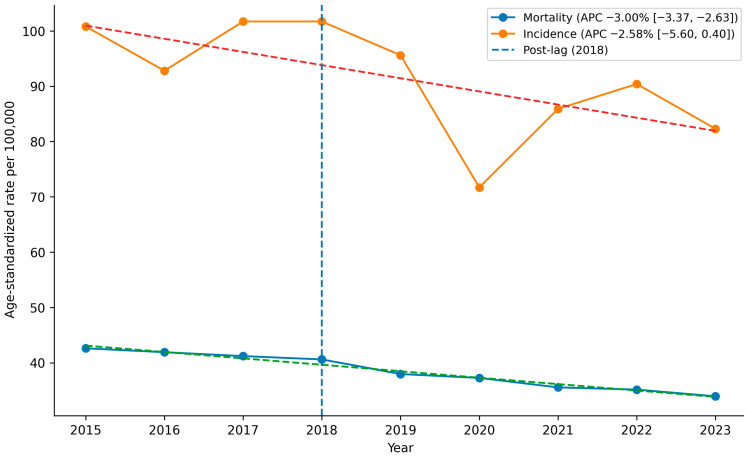
Global temporal trend of age-standardised colorectal cancer mortality and incidence rates in Galicia (2015–2023) with annual percent change (APC) estimates. Annual percent changes (APC) were estimated using log-linear regression models fitted to age-standardised rates over the full study period (2015–2023). APC and 95% confidence intervals were calculated from model coefficients using APC = (e^β^ − 1) × 100. Statistical significance was assessed using two-sided Wald tests.

**Figure 2:**
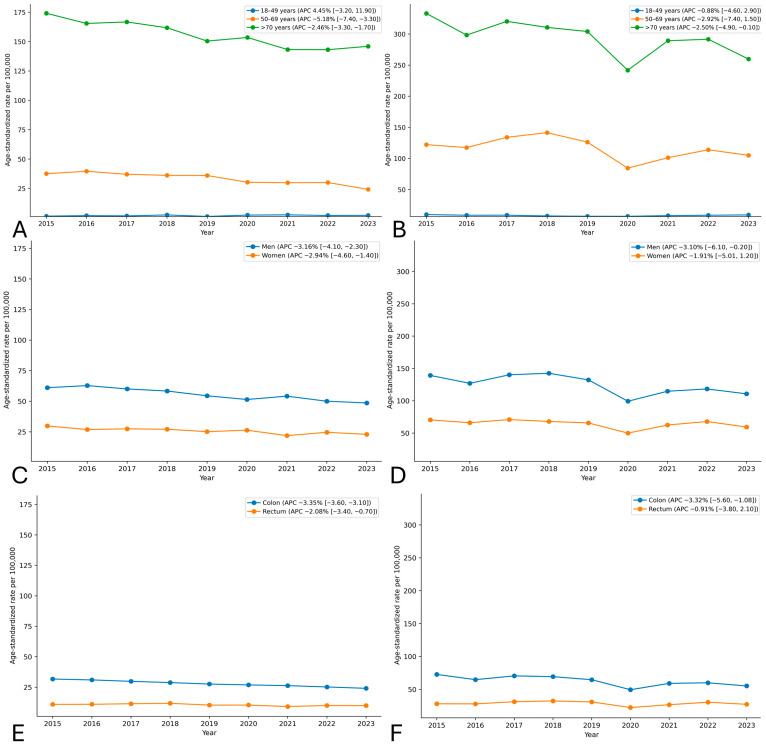
Temporal trend of age-standardised colorectal cancer mortality and incidence rates in Galicia (2015–2023) according to age, sex and tumour location with annual percent change (APC) estimates. (**A**) Mortality according to age groups; (**B**) Incidence according to age groups; (**C**) Mortality according to sex; (**D**) Incidence according to sex; (**E**) Mortality according to tumour location; (**F**) Incidence according to tumour location.

Across the full study period, independent of programme timing, overall CRC mortality showed a significant downward trend (APC −3.00%; 95% CI −3.37 to −2.63) (Table 2, Figure 1). A similar decline was observed for incidence (APC −2.58%; 95% CI −5.60 to 0.40). The reduction in mortality was more pronounced in the screening-eligible population (APC −5.18%; 95% CI −7.40 to −3.30), whereas trends were attenuated in individuals aged ≥ 70 years and non-significant among those aged < 50 years (Table 2, Figure 2). These estimates describe the overall temporal evolution and provide contextual information for the segmented ITS findings.

Age-standardised mortality and incidence rates are presented per 100,000 inhabitants. Dashed lines represent fitted log-linear regression models used to estimate the overall annual percent change (APC) across the full 2015–2023 period. APC values and 95% confidence intervals were derived from regression coefficients (β) of the model ln(rate) = α + β × year. Statistical significance was assessed using two-sided Wald tests for β = 0. The vertical dashed line indicates the transitional year (2018) following programme implementation.

### 3.4. Absolute and Relative Changes in Age-Standardised Rates

Complementary pre–post comparisons (2015–2017 vs. 2019–2023) further contextualised the magnitude of observed changes (Table 3 and Table 4). The overall ASMR declined from 41.92 (95% CI 40.26 to 43.58) to 35.91 (95% CI 33.92 to 37.90) per 100,000 inhabitants, corresponding to an absolute reduction of −6.01 per 100,000 (95% CI −8.00 to −4.02) and a relative decrease of −14.33% (95% CI −17.53 to −11.06) (Table 3, Figure 1). Among individuals aged 50–69 years, the relative reduction reached −20.98% (95% CI −29.92 to −11.78). Reductions were observed in both men (−15.61%) and women (−13.74%). By tumour site, mortality declined more markedly for colon cancer (−15.67%) than for rectal cancer (−10.62%) (Figure 2).

The overall ASIR declined from 98.37 (95% CI 94.10 to 102.60) to 85.16 (95% CI 81.40 to 88.90) per 100,000 inhabitants (Table 4, Figure 1), corresponding to an absolute reduction of −13.20 per 100,000 (95% CI −21.49 to −4.87) and a relative decrease of −13.42% (95% CI −21.60 to −5.10). In individuals aged 50–69 years, the relative reduction was −14.78% (95% CI −25.58 to −3.61). As with mortality, declines were more pronounced for colon than rectal cancer (Figure 2).

### 3.5. Participation and Exploratory Analysis

First-round participation ranged from 36.9% to 50.9% across healthcare areas. No statistically significant correlation was observed between participation rates and post-implementation mortality APC in the 50–69-year cohort (Spearman’s ρ = 0.429; *p* = 0.337) (Supplementary Figure S3).

## 4. Discussion

In this population-based study evaluating the implementation of an organised biennial FIT-based CRC screening programme in Galicia, we observed a modification in mortality trends following programme roll-out, particularly in the screening-eligible population aged 50–69 years. ITS analysis showed a reversal from increasing pre-implementation mortality slopes to sustained declines in the post-implementation period. Complementary analyses of age-standardised rates confirmed absolute and relative reductions in both mortality and incidence between the aggregated pre- and post-implementation periods. These effects were more pronounced in the target age group and for colon cancer, while no consistent trend reversal was observed in individuals younger than 50 years. Together, these findings support an association at the population level of organised FIT-based screening in a real-world setting and contribute to the limited body of evidence on the effectiveness—rather than efficacy—of contemporary CRC screening programmes.

The efficacy of CRC screening has been firmly established in randomised controlled trials (RCTs) and meta-analysis [14]. Classic guaiac-based faecal occult blood test (gFOBT) trials demonstrated reductions in CRC mortality ranging from 15% to 33% among participants, laying the foundation for organised screening strategies. Flexible sigmoidoscopy trials subsequently demonstrated reductions in both incidence and mortality, reinforcing the preventive potential of endoscopic screening.

More recently, pragmatic RCTs have added new evidence. The NordICC trial evaluating colonoscopy versus no screening reported a reduction in CRC incidence but only modest mortality effects at 10 years in intention-to-screen analysis, strongly influenced by participation rates [15]. Similarly, the Swedish SCREESCO trial comparing colonoscopy, FIT, and usual care demonstrated stage shift and increased early detection, although mortality results require longer follow-up [16]. The Spanish COLONPREV trial confirmed comparable detection performance between colonoscopy and FIT strategies [17]. While these RCTs establish screening efficacy under controlled conditions, they do not directly address how organised programmes perform at the population level once scaled within public health systems, where participation, adherence, and programme organisation become critical determinants of effectiveness.

Despite widespread adoption of FIT as the primary screening modality, robust evaluations of its real-world population effectiveness remain comparatively scarce. Several observational studies have suggested substantial mortality reductions in organised settings. Zorzi et al. reported significant mortality declines associated with FIT-based screening in Italy [6,18]. Levin et al., analysing Kaiser Permanente data, demonstrated marked reductions in CRC mortality following organised FIT implementation [8]. Similarly, Su et al. described reductions in incidence and mortality following programme introduction in a large community-based population [9]. Across Europe, however, direct comparison of programme effects remains challenging, as organised CRC screening strategies differ substantially in uptake, programme maturity, implementation model, and healthcare system context. As a result, variability in effectiveness likely reflects not only the screening modality itself but also differences in participation, adherence, and system-level organisation. In Spain, comparable population-level evidence remains scarce despite broad regional implementation of organised FIT-based programmes. Recent data from the Basque Country suggest a reduction in CRC mortality after long-term programme exposure, but published effectiveness analyses at population level are still limited [19]. In this context, the magnitude and pattern of change observed in Galicia appear consistent with those reported in well-organised European programmes with sustained uptake, although direct quantitative comparisons remain limited.

However, much of the published evidence reflects programmes that initially relied on guaiac-FOBT before transitioning to FIT, complicating attribution of observed effects specifically to FIT-based strategies. A recent systematic review and meta-analysis [11] highlighted that relatively few evaluated programmes were exclusively FIT-based from inception. Furthermore, heterogeneity limits comparability across settings. In this context, our findings provide additional population-level evidence from a region-wide organised FIT programme implemented within a unified public health framework. By applying interrupted time-series methods with area-specific roll-out definitions and latency assumptions, we attempted to disentangle secular trends from structural changes attributable to screening introduction. In this context, real-world evidence on the population-level effectiveness of contemporary FIT-based screening programmes remains comparatively limited, particularly in European settings, reinforcing the relevance of the present findings.

Unlike simple pre–post comparisons, ITS analysis allows formal modelling of pre-existing trends and estimation of slope changes following intervention introduction. This quasi-experimental approach is recommended when randomisation is not feasible [20,21]. By modelling pre- and post-implementation slopes separately and estimating ΔAPC, our study provides a measure of structural modification rather than mere temporal coincidence. The absence of comparable changes in the <50-year cohort strengthens causal plausibility.

Observed differences in the magnitude of incidence and mortality reductions according to sex are consistent with patterns previously described in population-based colorectal cancer screening programmes. In our study, reductions appeared more pronounced in men than in women. This finding is biologically and epidemiologically plausible and has been repeatedly observed in FIT-based screening settings, where men tend to have higher faecal haemoglobin concentrations, higher positivity rates, and higher detection rates for advanced neoplasia compared with women. As a result, FIT-based programmes may achieve greater early detection efficiency in men under a single threshold strategy [22,23]. Large population studies from organised screening programmes have consistently reported higher detection rates and positivity among men, as well as sex-related differences in interval cancer proportions and screening sensitivity. For example, analyses from the Basque population screening programme demonstrated substantially higher positivity and advanced neoplasia detection rates in men compared with women across different FIT thresholds [24]. Similarly, evaluations of the Scottish FIT programme have shown that interval cancer proportions are higher in women, suggesting lower test sensitivity when a single faecal haemoglobin threshold is applied to both sexes [23].

Differences between colon and rectal cancers should be interpreted cautiously. Most studies evaluating the effectiveness of colorectal cancer screening analyse outcomes according to proximal versus distal colon, rather than separating rectal cancer as an independent subsite. These subsite-specific analyses reflect known biological and epidemiological differences along the colorectum and have shown that screening effectiveness may vary by tumour location, particularly between proximal and distal disease [25]. In our study, registry data did not allow a robust stratification distinguishing distal colon from rectal cancers within the analytical framework used. Therefore, although reductions in incidence and mortality appeared smaller for rectal cancer, the mechanisms underlying this pattern remain uncertain. Earlier symptomatic presentation of rectal tumours may partly contribute, but current evidence specifically addressing rectal cancer within population screening programmes remains limited [26].

Participation is a key determinant of screening effectiveness. In our study, an exploratory area-level analysis did not identify a statistically significant association between first-round participation and changes in mortality trends. However, this analysis was limited by the small number of healthcare areas, and therefore lacked statistical power. These findings should not be interpreted as evidence of absence of effect, and participation likely remains a critical driver of programme impact.

Several strengths reinforce the interpretability of our results. We analysed consolidated population-based registry data covering the entire Autonomous Community, ensuring complete capture of CRC mortality and incidence. The asynchronous implementation across healthcare areas provided natural variation in intervention timing, strengthening quasi-experimental leverage. Age-stratified analyses, including individuals younger than screening age, functioned as internal negative controls. Moreover, the combination of segmented ITS, overall secular trend estimation, and complementary pre–post comparisons allowed triangulation of findings rather than reliance on a single analytical framework.

Nonetheless, several limitations merit consideration. First, this was an ecological population-level analysis; individual-level linkage between screening participation, adherence, and outcomes was not available. Consequently, causal attribution cannot be considered definitive. Although the <50-year cohort functioned as an internal negative control, the absence of an external contemporaneous comparison region limits the ability to fully exclude residual confounding by unmeasured temporal factors.

Second, improvements in CRC management—including advances in surgical techniques, perioperative care, and systemic therapies—may have contributed to mortality reductions during the study period. In addition, within the Spanish healthcare context, FIT has been incorporated into the diagnostic pathway for symptomatic patients to prioritise access to colonoscopy, as recommended in national clinical practice guidelines [27]. While this may have facilitated earlier diagnosis in some cases, these factors would be expected to produce more gradual and non-specific temporal changes. In contrast, the observed structural reversal of pre-existing upward trends and the age-specific concentration of effects within the screening-eligible population argue against purely treatment-driven explanations. Notably, although a reduction in mortality was also observed in individuals aged ≥ 70 years, this group includes individuals who may have previously participated in the screening programme before ageing out of the target population. Therefore, part of the observed improvement in this age group may reflect a cohort effect related to prior screening exposure rather than changes occurring exclusively in a never-screened population.

Third, the interpretation of segmented trends warrants caution. The relatively short time series, particularly in some healthcare areas, may limit the stability of slope estimates, and changes in slope (ΔAPC) should therefore be interpreted as descriptive indicators rather than precise effect estimates. In addition, pre-implementation increases in incidence and mortality may reflect artefacts related to registry maturation or changes in case ascertainment, potentially inflating baseline trends. External factors, including the COVID-19 pandemic, may also have influenced incidence patterns during the later study period [28]. Finally, the use of a fixed latency period and the absence of formal modelling of autocorrelation represent methodological simplifications inherent to the available data.

Finally, Galicia has a relatively homogeneous population and a unified regional public health structure, which may favour internal consistency of programme delivery but may limit extrapolation to more heterogeneous populations and healthcare systems.

Our findings suggest that organised FIT-based screening can be associated with measurable population-level reductions in CRC mortality within a relatively short post-implementation timeframe. The magnitude of effect appears concentrated in the screening-eligible age group, consistent with biological plausibility and trial evidence. However, we also observed mortality reductions in older age groups not actively invited to screening during the study period. This pattern may reflect a carry-over effect of prior screening exposure, whereby individuals previously invited within the eligible age range continue to benefit after ageing out of the programme. Such cohort effects have been described in long-term follow-up of organised screening programmes and may contribute to sustained population-level mortality reductions beyond the actively screened age band [9].

Future research should focus on longer follow-up, stage distribution dynamics, overdiagnosis assessment, participation optimisation, and comparative evaluations across regions with differing programme performance. Integration with cost-effectiveness modelling and health equity analyses will be essential to inform policy decisions.

## 5. Conclusions

In summary, in a real-world public health context, implementation of an organised FIT-based CRC screening programme was associated with a structural reversal in mortality trends and meaningful reductions in age-standardised rates. These findings contribute to the evolving evidence on the effectiveness of organised CRC screening beyond controlled trial settings and support continued optimisation of programme delivery at population level.

## Figures and Tables

**Table 1:** Interrupted time-series analysis: Annual percentage change (APC) before and after implementation and net change in trend (ΔAPC) in colorectal cancer mortality and incidence in Galicia.

Cohort	Mortality APC PRE (%) (95% CI)	Mortality APC POST (%) (95% CI)	Δ Mortality (%) (POST–PRE) *	Incidence APC PRE (%) (95% CI)	Incidence APC POST (%) (95% CI)	Δ Incidence (%) (POST–PRE) *
**Global**	13.70 (10.12, 17.39)	−3.62 (−4.47, −2.76)	−17.32	15.26 (5.48, 25.95)	−2.48 (−5.29, 0.41)	−17.74
**Sex**						
**Men**	17.59 (11.35, 24.17)	−3.40 (−5.20, −1.56)	−20.99	13.76 (4.65, 23.66)	−3.30 (−6.11, −0.41)	−17.07
**Women**	7.55 (−1.54, 17.49)	−3.41 (−5.47, −1.31)	−10.97	14.61 (−0.18, 31.59)	−1.59 (−5.12, 2.07)	−16.20
**Age**						
**≥70 years**	20.35 (14.42, 26.60)	−2.22 (−3.40, −1.02)	−22.57	20.64 (10.66, 31.53)	1.09 (−3.89, 6.33)	−19.55
**50–69 years**	11.80 (1.86, 22.72)	−8.08 (−10.43, −5.66)	−19.88	21.32 (4.60, 40.71)	−3.74 (−7.62, 0.30)	−25.06
**18–49 years**	11.56 (−51.04, 154.41)	−1.44 (−8.10, 5.71)	−13.00	1.60 (−34.03, 56.41)	0.72 (−6.67, 8.69)	−0.88
**Location**						
**Colon**	12.35 (4.18, 21.15)	−2.60 (−4.51, −0.65)	−14.94	14.41 (2.53, 27.67)	−1.69 (−5.15, 1.90)	−16.10
**Rectum**	12.71 (2.12, 24.39)	−2.49 (−5.34, 0.45)	−15.19	12.58 (1.31, 25.11)	−1.37 (−5.10, 2.50)	−13.96

APC values were estimated using segmented log-linear regression models of the form ln(rate) = β_0_ + β_1_(time) + β_2_(post) + β_3_(time × post). Pre- and post-implementation slopes were derived from model coefficients and transformed as APC = (e^β^ − 1) × 100. * The net change in trend (ΔAPC) was calculated as the difference between the post-implementation APC and the pre-implementation APC. As this measure derives from the difference between two correlated estimates, it is presented as a descriptive indicator and was not accompanied by confidence intervals.

**Table 2:** Pre–post variation and annual percent change (APC) in colorectal cancer mortality in Galicia, stratified by cohorts.

	CRC Mortality				CRC Incidence			
Cohort	Pre–Post Variation (%)	*p* ^1^	APC (%)	*p* ^1^	Pre–Post Variation (%)	*p* ^1^	APC (%)	*p* ^1^
**Global**	−14.33 (−17.37, −10.95)	<0.001	−3.00 (−3.37, −2.63)	<0.001	−13.42 (−21.60, −5.02)	<0.001	−2.58 (−5.60, 0.40)	0.07
**Sex**								
**Men**	−15.61 (−19.46, −12.01)	<0.001	−3.16 (−4.10, −2.30)	<0.001	−15.06 (−22.75, −7.10)	<0.001	−3.10 (−6.10, −0.20)	0.03
**Women**	−13.74 (−19.89, −6.97)	<0.001	−2.94 (−4.60, −1.40)	0.003	−11.61 (−20.08, −2.86)	<0.001	−1.91 (−5.01, 1.2)	0.1
**Age**								
**≥70 years**	−12.75 (−15.93, −9.82)	<0.001	−2.46 (−3.30, −1.70)	<0.001	−12.61 (−20.68, −4.52)	<0.001	−2.50 (−4.90, −0.10)	0.04
**50–69 years**	−20.98 (−29.92, −11.78)	<0.001	−5.18 (−7.40, −3.30)	<0.001	−14.78 (−25.58, −3.61)	<0.001	−2.92 (−7.40, 1.50)	0.1
**18–49 years**	23.51 (−5.93, 53.12)	0.12	4.45 (−3.20, 11.90)	0.2	−11.89 (−21.81, −1.40)	0.02	−0.88 (−4.60, 2.90)	0.6
**Location**								
**Colon**	−15.67 (−19.94, −11.34)	<0.001	−3.35 (−3.60, −3.10)	<0.001	−16.82 (−24.50, −8.49)	<0.001	−3.32 (−5.60, −1.08)	0.02
**Rectum**	−10.62 (−15.05, −6.80)	<0.001	−2.08 (−3.40, −0.70)	0.02	−5.32 (−15.04, 4.74)	0.3	−0.91 (−3.8, 2.10)	0.5

Pre–post variation compares mean age-standardised rates between 2015 and 2017 and between 2019 and 2023. Differences were calculated using arithmetic means, and 95% confidence intervals (95% CI) were estimated using parametric methods based on the t-distribution. APC values were estimated using log-linear regression across the full 2015–2023 period. Statistical significance was assessed using two-sided Wald tests. *p* ^1^: Differences considered statistically significant at *p* < 0.05.

**Table 3:** Age-standardised mortality rates (ASMR). Comparison between 2015 and 2017 and between 2019 and 2023.

Stratum	ASMR 2015–2017 (95% CI)	ASMR 2019–2023 (95% CI)	Absolute Change (95% CI)	Relative Change (%) (95% CI)
**Overall**	41.92 (40.26, 43.58)	35.91 (33.92, 37.90)	−6.01 (−8.00, −4.02)	−14.33% (−17.53, −11.06)
**Sex**				
**Men**	61.33 (57.90, 64.80)	51.75 (49.90, 53.60)	−9.58 (−11.92, −7.17)	−15.61% (−19.33, −11.80)
**Women**	28.06 (25.90, 30.20)	24.21 (23.10, 25.30)	−3.86 (−5.85, −1.95)	−13.74% (−20.08, −7.17)
**Age**				
**18–49 years**	1.64 (1.32, 1.96)	2.03 (1.65, 2.42)	0.39 (−0.11, 0.81)	23.51% (−5.93, 53.12)
**50–69 years**	38.06 (35.20, 40.90)	30.08 (28.40, 31.80)	−7.98 (−11.41, −4.48)	−20.98% (−29.92, −11.78)
**≥70 years**	168.78 (162.50, 175.00)	147.26 (143.10, 151.40)	−21.52 (−27.42, −16.37)	−12.75% (−15.93, −9.82)
**Location**				
**Colon**	30.83 (29.10, 32.60)	26.00 (24.90, 27.10)	−4.83 (−6.26, −3.42)	−15.67% (−19.94, −11.34)
**Rectum**	11.09 (10.30, 11.90)	9.92 (9.30, 10.60)	−1.18 (−1.66, −0.75)	−10.62% (−14.82, −6.87)

Mean ASMR values were calculated for 2015–2017 and 2019–2023. Absolute and relative changes were computed directly from period means. Ninety-five percent confidence intervals (95% CI) were estimated using parametric methods based on the t-distribution.

**Table 4:** Age-standardised incidence rates (ASIR). Comparison between 2015–2017 and between 2019–2023.

Stratum	ASIR 2015–2017 (95% CI)	ASIR 2019–2023 (95% CI)	Absolute Change (95% CI)	Relative Change (%) (95% CI)
**Overall**	98.37 (94.10, 102.60)	85.16 (81.40, 88.90)	−13.20 (−21.49, −4.87)	−13.42% (−21.60, −5.10)
**Sex**				
**Men**	135.50 (129.80, 141.30)	115.09 (109.70, 120.50)	−20.41 (−31.42, −8.91)	−15.06% (−22.74, −6.84)
**Women**	69.14 (65.20, 73.10)	61.12 (57.30, 64.90)	−8.02 (−14.02, −2.35)	−11.61% (−20.27, −3.47)
**Age**				
**18–49 years**	9.13 (8.20, 10.10)	8.04 (7.30, 8.80)	−1.09 (−2.05, −0.16)	−11.89% (−21.71, −1.82)
**50–69 years**	124.59 (118.70, 130.50)	106.18 (100.90, 111.40)	−18.41 (−32.84, −4.35)	−14.78% (−25.58, −3.61)
**≥70 years**	317.37 (302.50, 332.20)	277.36 (265.20, 289.50)	−40.01 (−67.20, −14.49)	−12.61% (−20.57, −4.76)
**Location**				
**Colon**	69.33 (65.70, 72.90)	57.67 (54.10, 61.20)	−11.66 (−17.52, −5.80)	−16.82% (−24.53, −8.70)
**Rectum**	29.04 (27.20, 30.90)	27.50 (25.80, 29.20)	−1.55 (−4.84, 1.58)	−5.32% (−16.59, 5.44)

Mean ASMR values were calculated for 2015–2017 and 2019–2023. Absolute and relative changes were computed directly from period means. Ninety-five percent confidence intervals (95% CI) were estimated using parametric methods based on the t-distribution.

## Data Availability

The data that support the findings of this study are derived from the Galician Tumour Registry (REGAT) and the Galician Health Service administrative databases. Access to these data is subject to institutional approval and data protection regulations and is therefore not publicly available. Aggregated data used in the analyses may be made available from the corresponding author upon reasonable request and with permission of the data providers.

## Supplementary Materials

The following supporting information can be downloaded at: https://www.mdpi.com/article/10.3390/cancers18081313/s1, Table S1. Area-specific implementation timeline and definition of analytical periods for interrupted time series analysis. Table S2. Interrupted time series analysis by healthcare area: pre- and post-implementation anual percentage change (APC) estimates for colorectal cancer mortality and incidence (overall and 50–69 years). Figure S1. Comparison of annual percent change (APC) pre- and post-implementation in colorectal cancer mortality by cohort (95%CI). Figure S2. Comparison of annual percent change (APC) pre- and post-implementation in colorectal cancer incidence by cohort (95%CI). Figure S3. Association between first-round participation in the Galician Colorectal Cancer Early Detection Programme and post-implementation mortality APC in the 50–69-year cohort.

## Abbreviations

The following abbreviations are used in this manuscript:

APC	Annual Percent Change
ASIR	Age-Standardised Incidence Rate
ASMR	Age-Standardised Mortality Rate
CI	Confidence Interval
CRC	Colorectal Cancer
FIT	Faecal Immunochemical Test
gFOBT	Guaiac Faecal Occult Blood Test
ITS	Interrupted Time Series
PGDPCC	Galician Colorectal Cancer Early Detection Programme (Programa Galego de Detección Precoz do Cancro Colorrectal)
REGAT	Galician Tumour Registry
RCT	Randomised Controlled Trial
