# Supplementary material for: Organised Colorectal Cancer Screening and Changes in Mortality and Incidence Trends: A Population-Based Study
Source: Cancers (Basel). 2026 Apr 21;18(8):1313. doi: 10.3390/cancers18081313 (PMC13114968; doi:10.3390/cancers18081313)
Supplement: Supplementary file 1 [file cancers-18-01313-s001.zip › cancers-4240976-supplementary.pdf]

cancers

MDPI

Supplementary material

Supplementary table S1. Area-specific implementation timeline and definition of analytical periods for interrupted time series analysis.

Supplementary table S2. Interrupted time series analysis by healthcare area: pre- and post-implementation anual percentage change (APC) estimates for colorectal cancer mortality and incidence (overall and 50–69 years)

Supplementary figure S1. Comparison of annual percent change (APC) pre- and post-implementation in colorectal cancer mortality by cohort (95%CI)

Supplementary figure S2. Comparison of annual percent change (APC) pre- and post-implementation in colorectal cancer incidence by cohort (95%CI)

Supplementary figure S3. Association between first-round participation in the Galician Colorectal Cancer Early Detection Programme and post-implementation mortality APC in the 50–69-year cohort

Table S1. Area-specific implementation timeline and definition of analytical periods for interrupted time series analysis.

Healthcare area	Pre-implementation period	Implementation phase	Post-implementation period*
Ferrol	Not available†	2013	≥2015
Ourense	Not available†	2015–2016	≥2017
Pontevedra	2015	2016–2017	≥2018
Lugo	2015–2016	2017–2018	≥2018
A Coruña	2015–2017	2018–2019	≥2019
Santiago	2015–2016	2017–2018	≥2018
Vigo	2015–2017	2018–2019	≥2019

* Post-implementation period defined after a two-year latency period following programme initiation.

† Pre-implementation period not available within the 2015–2023 observation window.

Table S2. Interrupted time series analysis by healthcare area: pre- and post-implementation anual percentage change (APC) estimates for mortality and incidence (overall and 50–69 years).

Healthcare area	CRC mortality APC (%) overall (95% CI)		CRC mortality APC (%) 50–69 (95% CI)		CRC incidence APC (%) overall (95% CI)		CRC incidence APC (%) 50–69 (95% CI)	
**Period**	**PRE**	**POST**	**PRE**	**POST**	**PRE**	**POST**	**PRE**	**POST**
Ferrol*	NA	-4.21 (-8.43, 0.21)	NA	-6.48 (-15.40, 2.95)	NA	-2.76 (-6.13, 0.73)	ND	-3.25 (-9.31, 3.22)
Ourense*	NA	-2.76 (-4.27, -1.22)	NA	-2.12 (-5.93, 1.87)	NA	-1.22 (-3.99, 1.62)	ND	1.47 (-7.78, 11.65)
Pontevedra	2.37 (-7.62, 13.44)	-2.57 (-6.50, -1.53)	13.47 (-22.09, 65.31)	-3.45 (-14.15, 8.59)	4.19 (-21.02, 37.39)	-0.32 (-5.44, 5.09)	34.47 (-39.04, 196.53)	0.05 (-16.09, 19.27)
Lugo	7.75 (-14.59, 35.93)	-1.69 (-3.22, -0.13)	19.00 (-39.20, 133.99)	-10.68 (-19.65, -1.10)	17.75 (-12.27, 57.89)	-1.74 (-6.36, 3.10)	44.18 (-25.28, 178.62)	-1.99 (-16.76, 15.41)
A Coruña	17.95 (10.10, 26.37)	-6.48 (-7.39, -5.56)	4.72 (-25.65, 47.49)	-14.27 (-17.57, -10.85)	25.08 (0.09, 56.28)	-3.90 (-7.38, -0.29)	-3.22 (-38.78, 52.95)	-6.20 (-14.52, 2.94)
Santiago	14.80 (-6.28, 40.65)	-4.59 (-6.99, -2.13)	26.53 (-29.01, 125.50)	-4.78 (-8.80, -0.59)	12.40 (-26.74, 72.43)	-1.87 (-7.69, 4.33)	44.56 (-31.86, 206.25)	-1.43 (-12.91, 11.57)
Vigo	20.29 (0.83, 43.50)	-1.69 (-3.22, -0.13)	12.61 (-47.75, 147.20)	-7.96 (-14.90, -0.46)	22.89 (-15.61, 79.01)	-1.31 (-6.54, 4.20)	29.06 (-49.75, 231.23)	-2.62 (-15.89, 12.74)
Weighted Galicia	13.70 (10.12, 17.39)	-3.63 (-4.45, -2.81)	11.80 (1.86, 22.72)	-8.08 (-10.57, -5.54)	15.26 (5.48, 25.95)	-2.49 (-5.33, 0.43)	-0.74 (-14.79,15.62)	-3.74 (-7.69, 0.38)

Area-specific APC estimates were derived from segmented log-linear interrupted time series models fitted separately for each healthcare area and cohort. APC was calculated from the slope coefficient (β) as APC = (e^β − 1) × 100. 95% confidence intervals (95% CI) correspond to the transformed standard errors of the slope estimates. Statistical inference was restricted to periods with at least three annual observations.

*In some healthcare areas, the pre-implementation period included only two years of observation. In these cases, pre-implementation APC estimates are presented descriptively, without statistical inference.

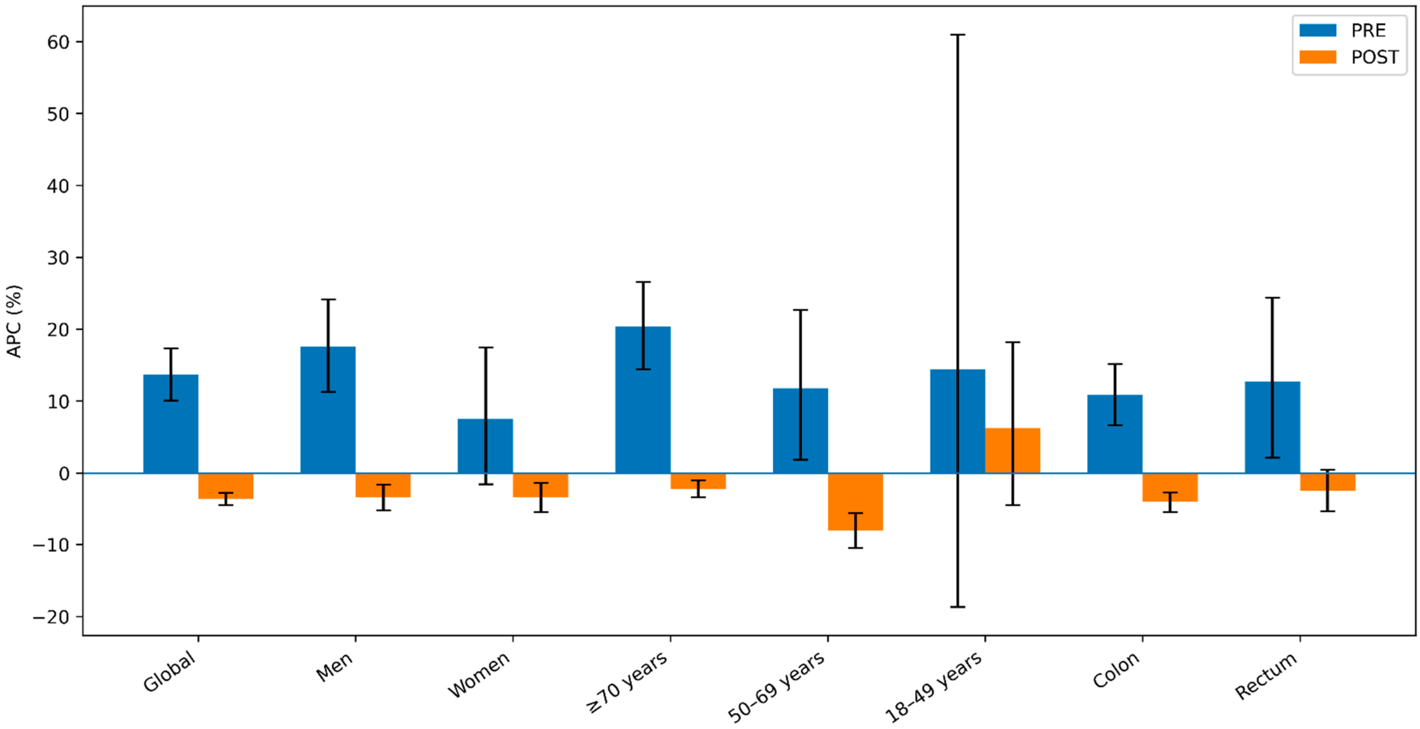

Figure S1. Comparison of annual percent change (APC) pre- and post-implementation in colorectal cancer mortality by cohort (95%CI). Segmented log-linear interrupted time series regression was used to estimate pre- and post-implementation APC. Confidence intervals correspond to slope parameters of the segmented model.

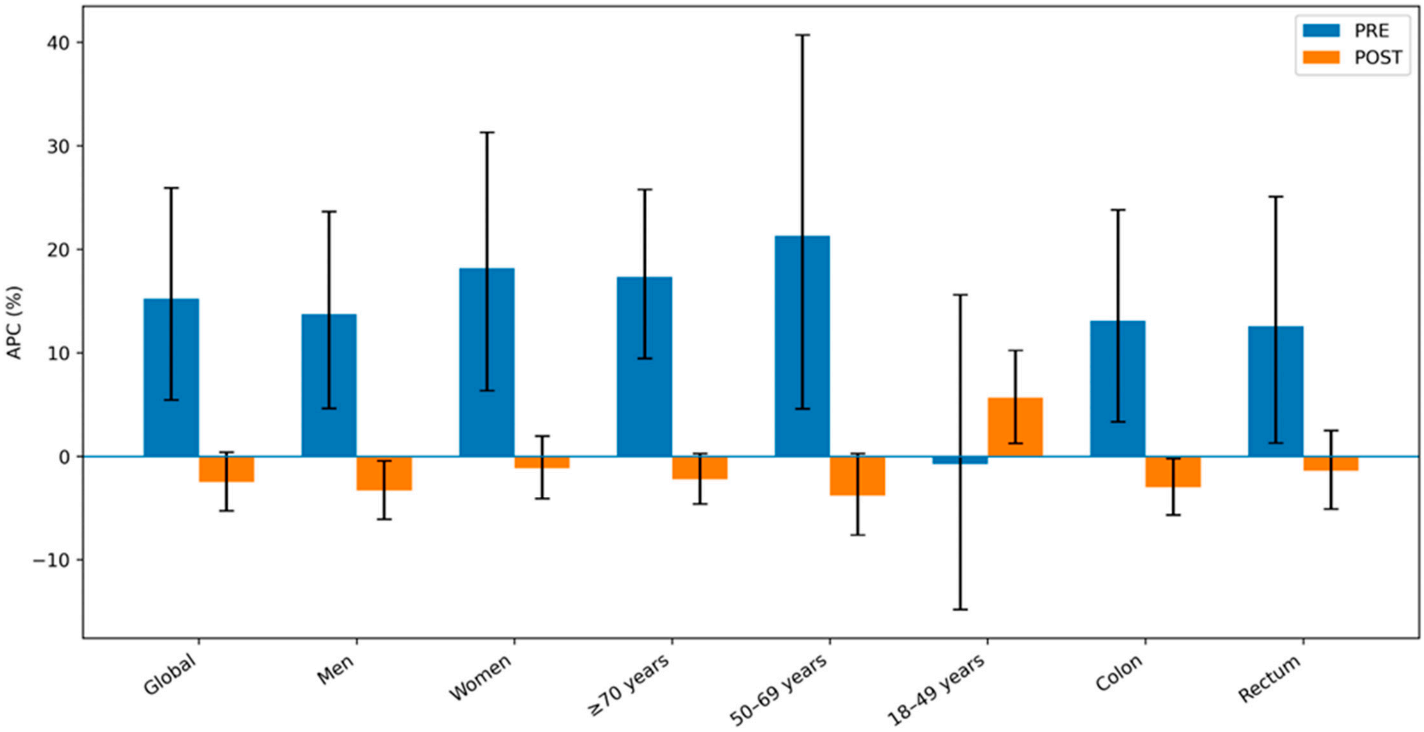

Figure S2. Comparison of annual percent change (APC) pre- and post-implementation in colorectal cancer incidence by cohort (95%CI). Segmented log-linear interrupted time series regression was used to estimate pre- and post-implementation APC. Confidence intervals correspond to slope parameters of the segmented model.

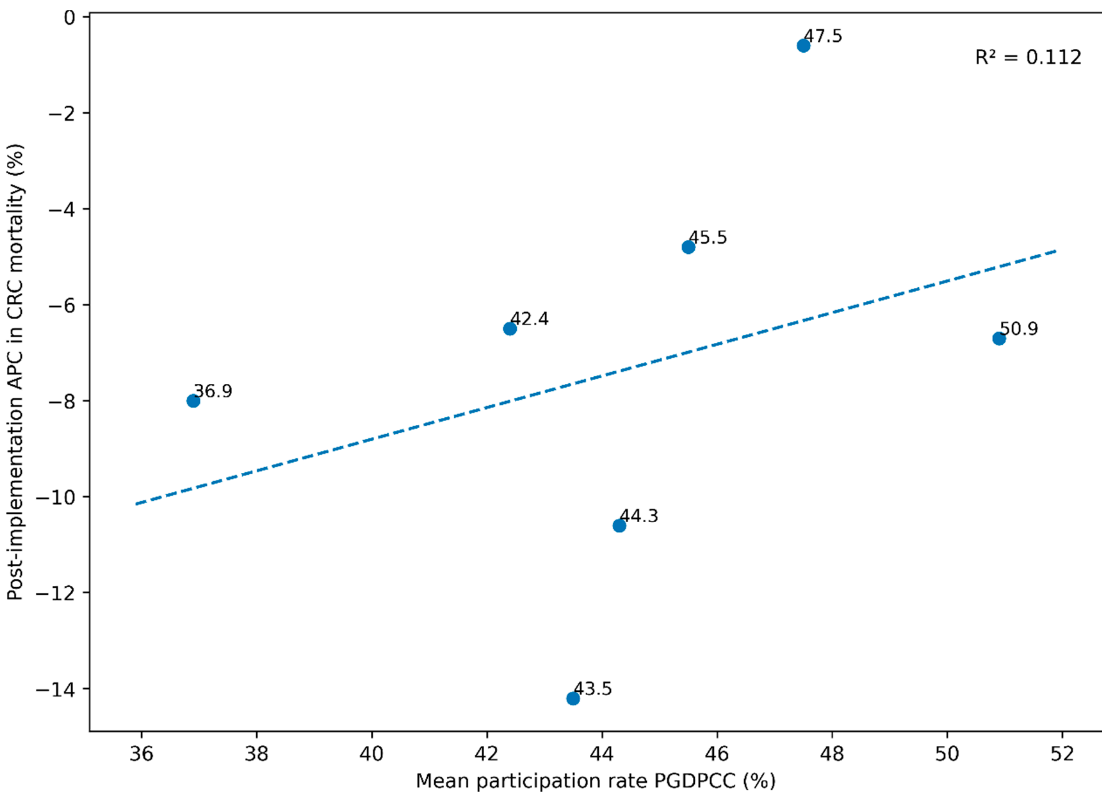

Figure S3. Association between first-round participation in the Galician Colorectal Cancer Early Detection Programme and post-implementation mortality APC in the 50–69-year cohort. Each point represents one healthcare area. The line corresponds to the least-squares linear fit. The association was evaluated using Spearman's rank correlation coefficient (two-sided test).
